# Microbubble Oxidation for Fe^2+^ Removal from Hydrochloric Acid Laterite Ore Leachate

**DOI:** 10.3390/ma16216951

**Published:** 2023-10-30

**Authors:** Ziyang Xu, Yu Wang, Boyuan Zhu, Guangye Wei, Fei Ma, Zhihui Yu, Jingkui Qu

**Affiliations:** 1 National Engineering Research Center of Green Recycling for Strategic Metal Resources, Institute of Process Engineering, Chinese Academy of Sciences, Beijing 100190, China; zyxu@ipe.ac.cn (Z.X.); byzhu@ipe.ac.cn (B.Z.);; 2 University of Chinese Academy of Sciences, Beijing 100049, China; 3 School of Chemistry and Materials Engineering, Liupanshui Normal University, Liupanshui 553004, China; wjtqb@126.com

**Keywords:** laterite, microbubble, hydrochloric acid lixivium, oxidation of Fe^2+^, oxygen mass transfer

## Abstract

After the atmospheric hydrochloric acid leaching method is used to treat laterite ore and initially purify it, the extract that results often contains a significant amount of Fe^2+^ impurities. A novel metallurgical process has been proposed that utilizes microbubble aeration to oxidize Fe^2+^ ions in laterite hydrochloric acid lixivium, facilitating subsequent separation and capitalizing on the benefits of microbubble technology, including its expansive specific surface area, negatively charged surface attributes, prolonged stagnation duration, and its capacity to produce active oxygen. The study examined the impacts of aeration aperture, stirring speed, oxygen flow rate, pH value, and reaction temperature. Under optimized experimental conditions, which included an aeration aperture of 0.45 µm, stirring at 500 rpm, a bubbling flow rate of 0.4 L/min, pH level maintained at 3.5, and a temperature range of 75–85 °C, the oxidation efficiency of Fe^2+^ surpassed 99%. An analysis of the mass transfer process revealed that microbubble aeration markedly enhances the oxygen mass transfer coefficient, measured at 0.051 s^−1^. The study also confirmed the self-catalytic properties of Fe^2+^ oxidation and conducted kinetic studies to determine an apparent activation energy of 399 kJ/mol. At pH values below 3.5, the reaction is solely governed by chemical reactions; however, at higher pH values (>3.5), both chemical reactions and oxygen dissolution jointly control the reaction.

## 1. Introduction

Nickel and cobalt are often associated with iron in laterite, a principal nickeliferous laterite mineral [1]. The extraction of valuable metals from this mineral involves methods such as reduction roasting–ammonia leaching [2,3], high-pressure acid leaching (AL) [4,5], and atmospheric AL [6,7,8]. Among these methods, atmospheric hydrochloric-acid-based AL is attracting increasing attention as an effective metal extraction process. In the AL process, iron is simultaneously extracted into the solution along with nickel and cobalt from the laterite ore. Fe^3+^ can be precipitated and separated from nickel–cobalt solutions by neutralization [9]. However, the separation of Fe^2+^ from nickel–cobalt solutions has proven difficult because of the phenomenon of coprecipitation upon neutralization. Before proceeding with additional processes like mixed hydroxide precipitation and the solvent extraction of nickel and cobalt, it is necessary to first oxidize and precipitate Fe^2+^ [10]. Nevertheless, when it comes to hydrometallurgy, the limited reactivity at low temperatures and the low solubility of atmospheric oxygen in aqueous solutions have constrained its direct utilization as an oxidizing agent in numerous applications.

Previous studies have reported an iron removal rate of 98.28% when iron was oxidized and removed from wastewater under specific conditions, which included a pH level of 6.92, an air flow rate of 500 mL/min, and a reaction time of 2.5 h [11]. However, it is important to note that in the context of laterite nickel ore leaching solutions, maintaining a pH level as high as 6.92 has been associated with significant losses of valuable elements such as nickel and cobalt, and the reaction kinetics have been observed to be relatively slow.

Under specific controlled conditions, SO_2_/O_2_ mixtures produce peroxymonosulfate (PMS) free radicals in solution, and these are a strong oxidant. The practical approaches necessary for enhancing the oxidation of Fe^2+^ using SO_2_/O_2_ at 75 °C were explored within the context of a process aimed at eliminating iron in the form of Fe^3+^ oxides from a simulated high-pressure acid leach solution of nickel laterite, maintained at a pH of around two [10]. However, the reaction product, SO_4_^2−,^ is considered an impurity in chloride systems.

The simultaneous removal of Fe^2+^ (2.0–4.2 mg/L) from groundwater was achieved through the application of PMS-assisted in situ oxidation/coagulation in combination with a ceramic ultrafiltration process [12]. Although this method has been reported to be capable of oxidizing Fe^2+^, it also has a few shortcomings. For instance, the oxidant is expensive, making this method more suitable for systems with a low iron content.

Fe^2+^ oxidation by O_2_ has been studied intensively by several researchers [13,14,15,16,17,18]. The rate of oxidation of Fe^2+^ was studied as a function of the pH, temperature, and ionic strength [16]. Furthermore, in prior research regarding the oxidation of neutral ferrous sulfate solutions, the rate equation was formulated in the following manner:

d[Fe^2+^]/dt = −k[Fe^2+^][OH^−^]^2^P_O2_ (1)

for the reaction

(2) $$Fe^{2+}+\frac{1}{4}O_{2}+2OH^{-}+\frac{1}{2}H_{2}O=Fe{\left(OH\right)}_{3}$$

where [OH^−^] = K_w_*/[H^+^], K_w_* is the stoichiometric dissociation constant for water [19]. The oxygenation of Fe^2+^ in neutral solutions is accelerated by the reaction product, iron hydroxide, and by the addition of iron hydroxide [20,21]. However, there have been few studies on the oxidation of ferrous ions in weakly acidic solutions (pH range of 3.3–4.7), despite the importance of this reaction with respect to preferential precipitation during hydrometallurgical processes.

Microbubbles are defined as bubbles with a diameter less than 50 µm and are valuable in various technical applications due to their characteristic behavior of reducing in size and collapsing when submerged in water [22]. Furthermore, they possess an extensive specific surface area, a surface with negative charge, prolonged stagnation capabilities, and a high efficiency in transferring oxygen [23]. Moreover, the collapse of microbubbles can generate free radicals, even without the presence of dynamic stimuli [22,24]. Therefore, the collapsing of air microbubbles results in the decomposition of substances like phenol and methyl orange [25,26]. Microbubbles have been recognized as suitable for a wide array of applications due to their exceptionally high bioactivity and efficiency in mass transfer. The concept of nucleating small bubbles as potential microbubbles through the compression of a gas stream for dissolution into a liquid and subsequent release via a specially designed nozzle system is based on the principles of cavitation [27]. The size of these microbubbles can be controlled by adjusting the pore size of the membrane employed [28].

In this study, a novel method of O_2_ microbubble-enhanced oxidation of Fe^2+^ was employed. Using the atmospheric pressure hydrochloric acid lixivium of laterite ore obtained from our team’s previous research as the raw material, the oxidation behavior of Fe^2+^ at lower pH values (approximately 3.3–4.7) was investigated, providing theoretical guidance for the subsequent one-step iron removal combined with the goethite method. The objectives of this study were to: (1) study the enhancement effect of microbubbles on the oxygen mass transfer process; (2) evaluate the ability of Fe^2+^ to undergo oxidation under different conditions; (3) and explore the kinetics of the reactions, including the sequential stages of oxygen dissolution and oxidation within the solution, with consideration given to the catalytic effects of ferric hydroxide.

## 2. Experimental

### 2.1. Samples and Methods Used

The experiments were carried out within a 1 L glass reactor with multiple necks, placed in a temperature-controlled water bath accurate to within ±1 °C. The reactor was loaded with 500 mL of a simulated solution of hydrochloric acid solution of laterite, containing 1 g/L of Fe^2+^, 0.048 g/L of Ni, 0.007 g/L of Co, 0.03 g/L of Mn, 0.097 g/L of Al, 0.045 g/L of Cr, and 6 g/L of Mg, and heated to the predetermined temperature. Pure compressed oxygen or air from a cylinder (Huanyujinghui, China) was introduced externally and directed through titanium microporous filters (Tianjian, China) into the solution. The pH of the solution was kept constant through automated titration using NaOH, while DO (dissolved oxygen) levels were continuously monitored throughout the experiment. To halt the oxidation reaction, liquid samples were promptly subjected to acidification. Subsequently, the solution was rapidly cooled, and ferrous ion analysis was performed. 

To investigate the kinetics of oxygen mass transfer, a simulated acid solution devoid of Fe^2+^ was created. Nitrogen (N_2_) was bubbled into the solution to eliminate dissolved oxygen. Subsequently, external oxygen (O_2_) was introduced and passed through titanium microporous filters into the solution. The dissolved oxygen (DO) content was then monitored over a specified duration.

All the reagents used were obtained from various suppliers and employed without further purification. The water used was deionized to minimize the traces of the dissolved metal ions and salts. All the experiments were repeated thrice, and the data shown are the means.

### 2.2. Analytical Method

The concentration of Fe^2+^ was assessed through titration, utilizing a standard K_2_Cr_2_O_7_ solution with Na-diphenylamine-sulfonic acid as an indicator [29]. The Fe^3+^ concentration was calculated by subtracting the Fe^2+^ concentration from the total iron concentration, determined using inductively coupled plasma optical emission spectrometry [30]. Dissolved oxygen (DO) levels were measured employing a DO meter (Seven2Go, Mettler, Switzerland).

## 3. Results and Discussion

### 3.1. Effect of Oxygen Mass Transfer by Microbubble Aeration

**Figure 1:**
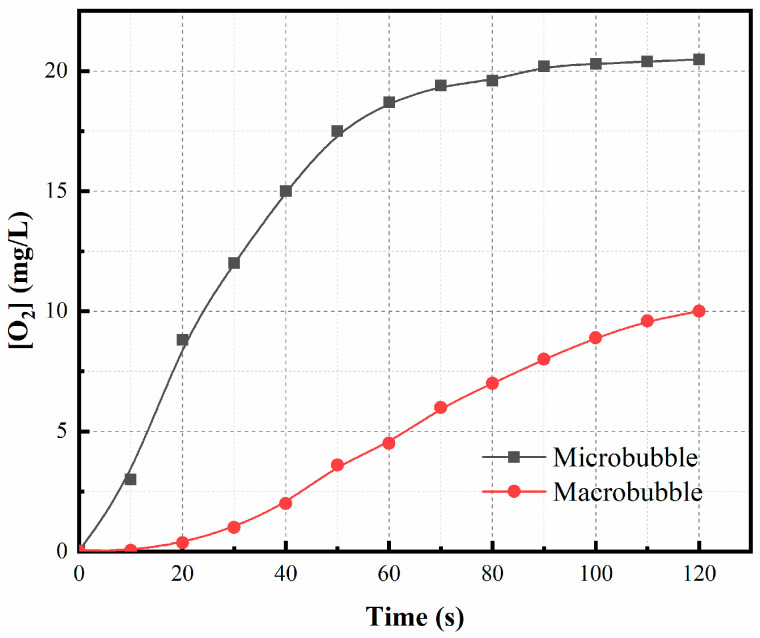
Dissolved oxygen (DO) concentration vs. time for different size bubbles. Experimental conditions: oxidation time = 8 min, temperature = 75 °C, bubbling flow = 0.4 L/min, pH = 3.5, stirring speed = 500 rpm.

Figure 1 depicts the changes in dissolved oxygen (DO) concentration in the simulated acid lixivium lacking Fe^2+^ over time at different aeration capacities. Initially, DO concentrations increased rapidly due to significant driving forces facilitating oxygen mass transfer, ultimately reaching a saturation point at 20.45 mg/L. It is worth noting that this saturation concentration is slightly lower than that observed in deionized water (22.17 mg/L). This discrepancy is attributed to the presence of electrolytes in the laterite hydrochloric acid solution, leading to a salting-out effect that diminishes oxygen solubility [31]. Subsequently, DO concentration exhibited a gradual increase during aeration with larger bubbles.

The relationship shown in Figure 1 fits the basic mass transfer equation

(3) $$\ln\left({\left[O_{2}\right]}_{s}-[O_{2}]\right)=-K_{La}t+ln\left({\left[O_{2}\right]}_{s}-{\left[O_{2}\right]}_{0}\right)$$

where [O_2_]_s_ is the oxygen concentration at saturation, [O_2_] is the oxygen concentration in the medium, [O_2_]_0_ is the oxygen concentration at time t = 0, K_La_ is the oxygen transfer coefficient, and t is the time.

**Figure 2:**
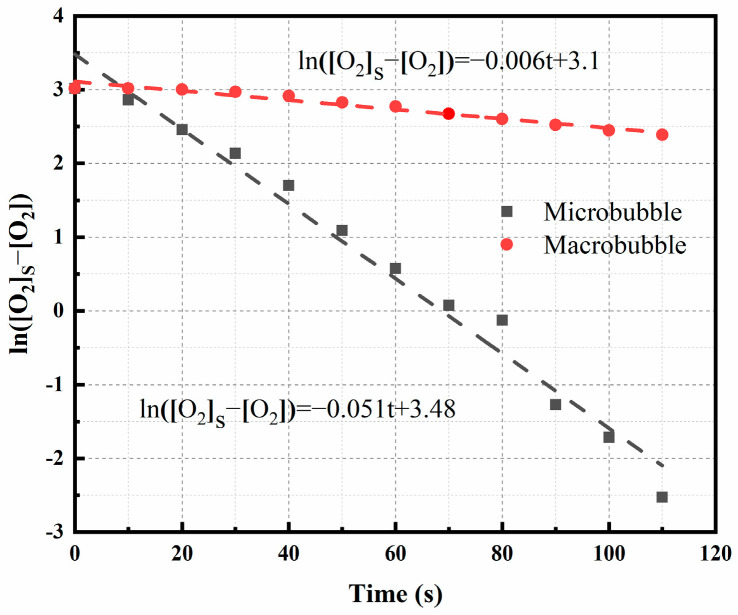
Relationship between ln([O_2_]_s_–[O_2_]_0_) and time.

The model’s plot takes the form of a linear graph, with the slope representing the oxygen transfer coefficient. Figure 2 illustrates the relationship between ln([O_2_]_s_–[O_2_]) and time in both macrobubble and microbubble scenarios. Notably, the oxygen mass transfer coefficient for microbubbles (0.051 s^−1^) surpassed that of macrobubbles (0.006 s^−1^) by a factor of 8.5. This remarkable difference can be attributed to the negatively charged surface properties of microbubbles, which hinder their aggregation [32] and enable them to maintain a substantial specific surface area. According to the Yang–Laplace equation [23], the small diameter of microbubbles results in a significant internal pressure. All these factors collectively enhance the rate of mass transfer from the bubbles into the aqueous phase, leading to a higher concentration of dissolved gas in the aqueous phase. Consequently, a higher oxygen mass transfer rate significantly enhances the efficiency of Fe^2+^ oxidation.

### 3.2. Effects of Experimental Conditions on Fe^2+^ Oxidation

#### 3.2.1. Effect of Aeration Aperture

**Figure 3:**
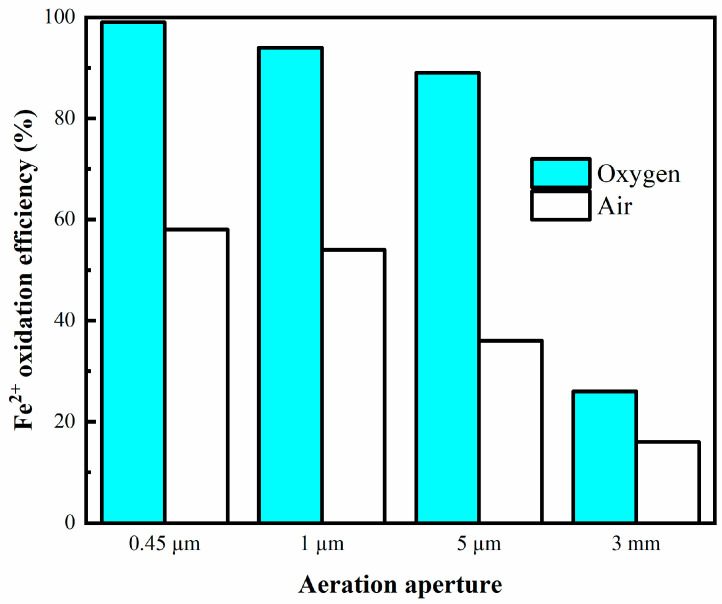
Oxidation efficiency of Fe^2+^ for different oxidation methods. Experimental conditions: oxidation time = 8 min, temperature = 75 °C, bubbling flow = 0.4 L/min, pH = 3.5, stirring speed = 500 rpm.

The effect of the aeration aperture on the enhanced oxidation of Fe^2+^ from the laterite hydrochloric acid lixivium was examined. The experimental results are shown in Figure 3. It can be seen that in the case of pure oxygen, the efficiency of oxidation using a 0.45 μm aeration head was very high, and the oxidation process was completed within 8 min. However, as the aperture of the aeration head was increased, the oxidation efficiency decreased gradually and was the lowest at 3 mm. This is due to the increase in the diameter of the aeration head, which led to an increase in the diameter of the bubbles generated [28]. This caused more bubbles to rise to the liquid level and then disappear. In addition, the mass transfer inside the liquid phase was reduced [33], and the production of active oxygen also decreased indirectly [22].

As shown in Figure 3, the efficiency of oxidation with pure oxygen and air was also compared. The oxygen concentration also affects the efficiency of oxidation of Fe^2+^; the higher the oxygen concentration, the faster the reaction rate. There are two possible reasons for this. On the one hand, the partial pressure of oxygen in the case of pure oxygen is greater than that in air, which enhances the mass transfer process. On the other hand, oxygen facilitates the formation of ·OH radicals, and consequently accelerates the oxidation process [25]. It has also been reported that reactive oxygen species, such as superoxide anion radicals, H_2_O_2_, and ·OH radicals, are generated during the reduction of molecular oxygen to water through the acceptance of four electrons [34].

#### 3.2.2. Effect of Stirring Speed

**Figure 4:**
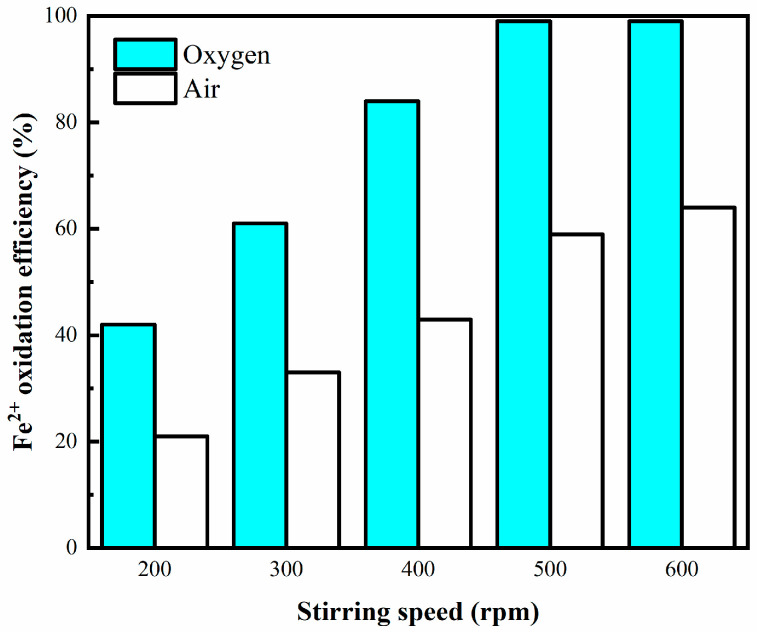
Oxidation efficiency of Fe^2+^ vs. stirring speed. Experimental conditions: oxidation time = 8 min, temperature = 75 °C, bubbling flow = 0.4 L/min, pH = 3.5, aeration aperture = 0.45 μm.

Figure 4 illustrates the impact of stirring speed on the enhanced oxidation of Fe^2+^. Following an 8 min oxidation period, it was observed that the efficiency of Fe^2+^ oxidation was notably higher when the stirring speed exceeded 500 rpm. This phenomenon can be attributed to the primary processes involved in bubble formation from the pores, which include bubble growth and detachment. When gas was introduced, microbubbles initiated their growth phase. Once the separation force generated by the water phase flow exceeded the retaining force, bubbles detached from the pore opening. Vigorous stirring amplified the separation force, resulting in smaller diameter bubbles [35]. Another valid reason is definitely the decrease in the thickness of diffusion mass transfer boundary layers on the surface of each bubble due to increase in stirring speed. This results in enhanced/faster mass transfer to the bulk [36,37]. Furthermore, an increase in mass transfer can also be attributed to the effect of turbulence within the medium, leading to a higher kinetic energy dissipation rate [38]. Consequently, an increase in stirring speed contributed to improved gas dispersion, thereby enhancing mass transfer.

#### 3.2.3. Effect of Bubbling Flow Rate

**Figure 5:**
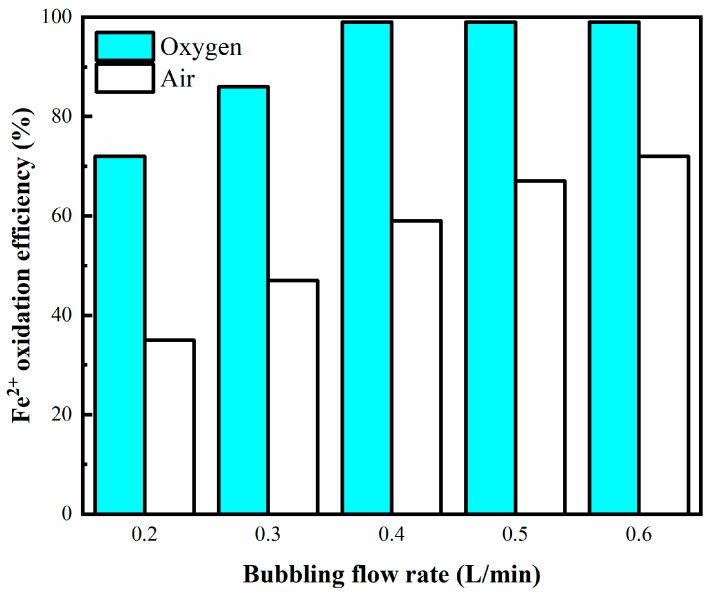
Oxidation ratio of Fe^2+^ vs. bubbling flow rate. Experimental conditions: oxidation time = 8 min, temperature = 75 °C, stirring speed = 500 rpm, pH = 3.5, aeration aperture = 0.45 μm.

Figure 5 illustrates that during the initial 8 min period, an increment in the flow rate led to a progressive rise in the efficiency of Fe^2+^ oxidation. When oxygen is supplied, the deep oxidation of Fe^2+^ can be achieved at a flow rate > 0.4 L/min, and the oxidation rate can be greater than 99%. Previous studies suggest that the mean bubble diameter is barely affected by the flow velocity or surface tension [37]. Therefore, for the same reaction time, an increase in the gas flow rate means that more microbubbles are generated; this enhances mass transfer and results in the production of more free radicals, thereby aiding the oxidation process.

#### 3.2.4. Effect of pH

**Figure 6:**
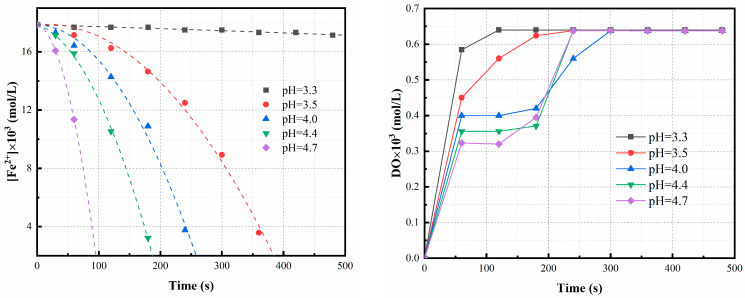
Experimental data (symbols) and predicted values (lines) for oxidation of Fe^2+^ at different pH values. Experimental conditions: oxidation time = 8 min, temperature = 75 °C, bubbling flow = 0.4 L/min, pH = 3.5, stirring speed = 500 rpm, aeration aperture = 0.45 μm.

As can be seen in Figure 6, the rate of oxidation of the Fe^2+^ increases with the increase in pH, and the change in the reaction rate with the pH can be explained based on the effect that hydroxyl ions has on the reaction rate [19]. Concurrently, H^+^ is the product of Fe^2+^ oxidation precipitation reaction; the fact that its concentration is lower means that the reaction proceeds to the right, thereby resulting in a higher reaction rate. According to the results of a previous experimental study [9], in the case of Ni laterite hydrochloric acid leaching liquors, the Ni recovery rate is affected by the balance between precipitation and dissolution and decreases as the pH is increased. Therefore, the optimal oxidation pH was determined to be 3.5.

#### 3.2.5. Effect of Temperature

**Figure 7:**
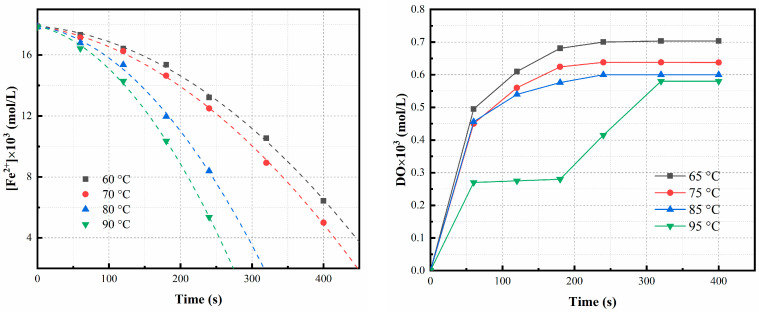
Experimental data (symbols) and predicted values (lines) for oxidation of Fe^2+^ at different temperatures. Experimental conditions: oxidation time = 8 min, bubbling flow = 0.4 L/min, pH = 3.5, stirring speed = 500 rpm, aeration aperture = 0.45 μm.

The impact of temperature on the rate of Fe^2+^ oxidation was also examined. As shown in Figure 7, in the temperature range of 65–95 °C, the oxidation ratio of Fe^2+^ increases with increasing temperature. The oxidation ratio of Fe^2+^ is primarily affected by the oxygen reaction activity, oxygen mass transfer coefficient, and DO concentration. According to the theory of kinetics, increasing the temperature can increase the percentage of activated molecules and the diffusion coefficient, and simultaneously decrease the oxygen solubility [6,39,40]. At low temperatures, the former dominates, promoting the generation of active oxygen species, increasing molecular collisions, and reducing activation energy. However, the decrease in the oxygen solubility becomes more pronounced at high temperatures, thereby inhibiting the formation of active oxygen. Conversely, the increase in the reactivity of the reactive species caused by the increase in the temperature is generally the main factor leading to a change in the oxidation rate of ferrous ions.

### 3.3. Study of Macroscale Kinetics

The effect of microbubble aeration as an enhanced method on the system is to disperse the gas, accelerate mass transfer, and promote the reaction; however, it also increases the activity of the reactants through the system’s violent collision, and reduces the threshold of reaction occurrence [14,41,42,43,44]. Because most conventional studies on Fe^2+^ oxidation have reported low reaction rates and used low Fe^2+^ concentrations, the DO concentrations can be considered approximately constant. However, the system is fully energized under the conditions of microbubble aeration, and its kinetics may be different from those of the conventional form.

In Figure 6, the time variation of Fe^2+^ concentration measured at different pH values is depicted. It can be observed that the first-order reaction curve of [Fe^2+^] exhibits a concave shape, deviating from the rate Equation (1). The oxidation clearly demonstrates autocatalytic properties, and as both the initial concentration and reaction time increase, it progressively diverges from normal first-order kinetics. The acceleration of oxidation may be attributed to the catalytic effect exerted by reaction products, which intensifies with ongoing oxidation [42].

**Figure 8:**
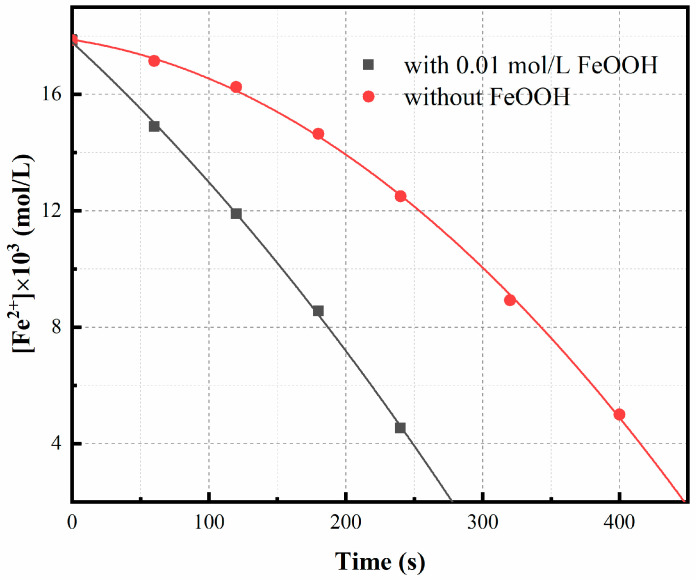
Effect of FeOOH on oxidation rate. Experimental conditions: oxidation time = 8 min, temperature = 75 °C, bubbling flow = 0.4 L/min, pH = 3.5, stirring speed = 500 rpm, aeration aperture = 0.45 μm.

To investigate the impact of heterogeneous reactions, we conducted an oxidation experiment: a 0.01 mol/L FeCl_2_ solution was subjected to oxidation for one hour at a temperature of 75 °C, pH value of 3.5, and rotation speed of 500 rpm, followed by filtration. Subsequently, the filtered residue was introduced into a simulated lixivium and further oxidized under identical experimental conditions. As depicted in Figure 8, it is evident that the presence of precipitate significantly enhances the rate of oxidation compared to its absence.

The oxidation of Fe^2+^ by dissolved oxygen (DO) takes place through two distinct pathways [20,21]: one involves a homogeneous reaction within the solution, while the other entails a heterogeneous reaction occurring on the precipitate. The latter term makes a more substantial contribution. Thus,

(4) $$Fe^{2+}+O_{2}={Fe}^{3+}+O_{2}^{-}\;\left(\mathrm{homo}\right)$$

(5) $$Fe_{ad}^{2+}+O_{2}={Fe}^{3+}+O_{2}^{-}\;\left(\mathrm{hetero}\right)$$

We made the assumption that the adsorption of Fe^2+^ onto the precipitate reaches equilibrium. The observed oxidation rate is the combined result of both homogeneous and heterogeneous reactions and, under a constant pH condition, can be expressed as

−d[Fe^2+^]/dt = k[Fe^2+^][O_2_] + k_s_[Fe^2+^][O_2_]L_1_ (6)

where k is the rate constant of the homogeneous reaction, k_s_ represents the rate constant for the heterogeneous reaction, and L_1_ denotes the concentration of adsorbed Fe^2+^. In a study conducted by Tamura et al. [20], the ratio L_1_/[Fe^2+^] was determined under varying pH levels and precipitate quantities, yielding the subsequent empirical equation

L_1_/[Fe^2+^] = K_1_L_p_/[H^+^] (7)

where L_p_ was the concentration of iron precipitate, and the constant K_1_ was 1.41 × 10^−5^ at 298 K.

Given the assumption that the precipitate’s surface characteristics in this study resemble those observed by Tamura et al. [20] and that their K_1_ value can be extended to our investigation, it can be concluded that L_1_ is notably lower than L_p_, and

L_p_ ≈ [Fe^2+^]_0_−[Fe^2+^] (8)

From Equations (6)–(8), we obtain

(9) $$-\frac{1}{\left[{Fe}^{2+}\right][O_{2}]}\frac{d\left[{Fe}^{2+}\right]}{dt}=k+k_{1}({\left[{Fe}^{2+}\right]}_{0}-\left[{Fe}^{2+}\right])$$

where

k_1_ = k_s_K_1_/[H^+^] (10)

**Figure 9:**
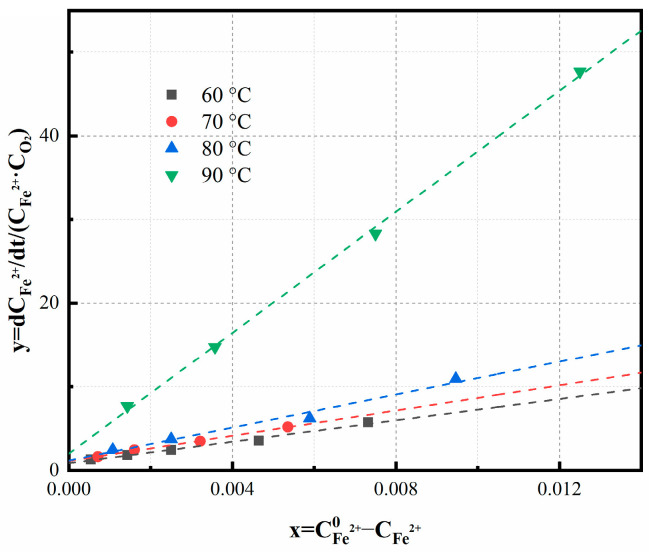
[Fe^2+^] and [O_2_]/[O_2_]_s_ vs. time. Experimental conditions: oxidation time = 8 min, temperature = 75 °C, bubbling flow = 0.4 L/min, stirring speed = 500 r/min, aeration aperture = 0.45 μm, pH = 3.5.

Using the experimental data for [Fe^2+^], [O_2_], and d[Fe^2+^]/dt, we plotted the left-hand side of Equation (9) against ([Fe^2+^]_0_−[Fe^2+^]) in Figure 9. The parameters ‘k’ and ‘k_1_’ were determined through linear regression analysis, with ‘k’ corresponding to the intercept, and ‘k_1_’ being derived from the slope of the linear regression.

**Figure 10:**
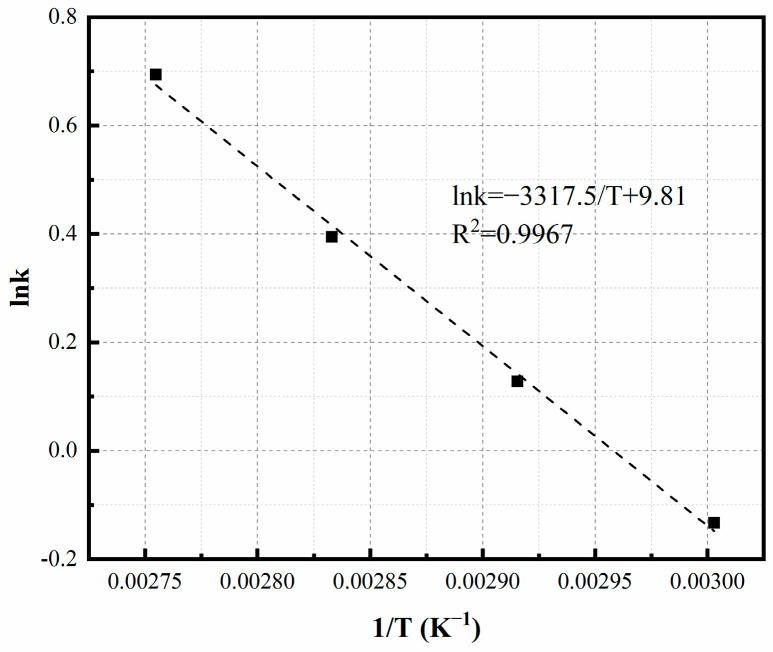
Temperature dependence of k. Experimental conditions: oxidation time = 8 min, bubbling flow = 0.4 L/min, pH = 3.5, stirring speed = 500 rpm, aeration aperture = 0.45 μm.

The relationship between temperature and the rate constant ‘k’ is presented in Figure 10, with the activation energy calculated to be 399 kJ/mol. The variations of Fe^2+^ ions and dissolved oxygen concentration over time under different pH conditions are illustrated in Figure 6. Initially, the oxidation reaction exhibits an accelerated rate, indicating that heterogeneous reactions play a significant role as precipitation increases. During the experimental conditions with a pH lower than 3.5, the dissolved oxygen concentration swiftly attains saturation upon the initiation of oxidation, indicating that chemical reaction primarily dictates the overall rate. Conversely, at elevated pH levels, the oxygen concentration remains below saturation, indicating that both the chemical reaction and the dissolved oxygen collectively influence the overall oxidation rate.

## 4. Conclusions

A comprehensive investigation has been undertaken in order to explore the optimization of Fe^2+^ oxidation in the hydrochloric acid leachate of laterite through the utilization of the microbubble aeration method. An analysis of the mass transfer process has unveiled that the oxygen mass transfer coefficient, K_La_ (0.051 s^−1^), associated with the microbubble aeration approach surpasses that of the macrobubble aeration method (0.006 s^−1^) by a substantial factor of 8.5. Single-factor experiments have demonstrated that under ideal conditions (including an aeration orifice diameter of 0.45 μm, a stirring speed of 500 rpm, a gas flow rate of 0.4 L/min, a pH level of 3.5, a temperature range of 75–85 °C, and an oxidation duration of 8 min), the efficacy of Fe^2+^ oxidation exceeds an impressive 99%. It has been substantiated that the microbubble oxygen exhibits inherent self-catalytic properties in the context of the Fe^2+^ oxidation reaction. Subsequent kinetic investigations have disclosed an apparent activation energy of 399 kJ/mol. It is important to note that the governing mechanism of the reaction primarily relies on chemical reactions when the pH is maintained below 3.5. However, when the pH surpasses the threshold of 3.5, both chemical reactions and oxygen dissolution act in concert to control the reaction.

## Data Availability

The data supporting the article’s findings are available from the corresponding author upon reasonable request.
